# Revisiting Happiness and Well-Being in Later Life from Interdisciplinary Age-Studies Perspectives

**DOI:** 10.3390/bs9090094

**Published:** 2019-09-03

**Authors:** Ieva Stončikaitė

**Affiliations:** Grup Dedal-Lit, University of Lleida, 25003 Lleida, Spain; iewukaz@yahoo.com

**Keywords:** happiness, well-being, successful ageing, quality of life, later life

## Abstract

Important demographic shifts and the so-called ‘longevity revolution’ have generated profound transformations in social interpretations of old age, an increased interest in age studies and new ideas on how to age well. The majority of current successful ageing models, however, represent rather a prevailing construct in Western societies. Physical and psychosocial well-being and the ability to adjust to the ideals of successful ageing are often seen as an integral part of a good quality in life. Those who do not or cannot follow these lines are often regarded as morally irresponsible and seem to be doomed to have a lonely, unhealthy and unhappy later life. This paper questions the current discourses of successful ageing in terms of healthy and happy living and calls for a reconsideration of more global, integrated and holistic understandings of the process of growing old.

## 1. Changing Social Demographics and Understandings of Old Age

According to UNFPA, the United Nations Population Fund (2012), there are more than 850 million people in the world over the age of 60, which is almost 12.3% of the global population [1]. In Europe alone, the total population is projected to grow from 511 million in 2016 to 520 million in 2070 [2]. In general, human longevity has increased by around 30 years, which constitutes almost a whole generation [3]. Although ageing is a global phenomenon, women, in comparison to men, tend to live longer, and, therefore, belong to the fastest growing group of the older population. Catherine B. Silver observes that “[s]ince the turn of the century, individuals in post-industrial societies have gained, on average, 30 years in life expectancy, women have gained 7 more years than men” [4] (p. 380). The increase in human longevity is due to many factors such as preventive medical assistance, improvements in hygiene, food supply and technology, higher standards of living, financial stability, funding for pensions and housing, immunizations, public health initiatives and social and economic developments [5,6]. 

At the biological level, ageing is often defined “by the accumulation of molecular damage that progressively leads to structural and functional abnormalities in cells, tissues and systems” [7] (p. 1). Yet, according to many gerontologists, ageing is not a simple and fixed chronological process that ends with death, but is a multifaceted and open-ended experience, conditioned by socio-cultural and historical elements [8,9]. As Mike Hepworth states, ageing “is not a straightforward linear trajectory towards inevitable physical, personal and social decline, but a dynamic process of highly variable change: ageing is simultaneously a collective human condition and an individualized subjective experience” [9] (p. 1). Along the same lines, Kathleen Woodward notes that age is a “fundamental and endlessly interesting category” that continues to permeate everyday experiences from the moment of birth to the end of our lives and gives meaning to our existence [10] (pp. 4–5). 

With an increase in human longevity, age-focused norms have become more blurred and more flexible. These changes give way not only to different interpretations and understandings of age, but also to the transcendence of age-related categories and behaviours [11,12,13]. Stephen Katz and Jessica Gish state that age, as a social category, is no longer valid in a culture in which social expectations and roles have become less defined: “we are as young, or as old, as we *feel* since age is no longer the only way to measure living in time” [14] (p. 40, emphasis in original). Chris Gilleard and Paul Higgs also note that “[a]geing is not what it once was” [12] (p. vii). While the generation previous to baby-boomers saw ageing as a normative life course trajectory, the postwar generation has aged in very different ways from those of their parents or grandparents. The 1960s was a time when Victorian moral prescriptions were challenged, which gave more flexibility to self-expression and new meanings of old age and ageing [12]. The ‘cultural break’ of the 1960s and the transition from popular to mass culture greatly affected the Western world “setting one generation against another” [12] (p. 26). The rebirth of ‘youth culture’ resulted in a “generational schism that set apart the ‘old’ and the ‘new’” [12] (p. 26). Yet, this generational gap was narrowed by a “combination of marketing, the new media and the postwar entertainment, leisure and self-care industries” [12] (p. x). Increased consumption and exposure to new market goods, fashion, the beauty ideals and self-care products and services led to the adoption of more diverse roles available to older people. These changes were visible in new ways of self-expression, personal choices and lifestyles, which were marked by more freedom and the invention of middle age lifestyles. In other words, the traditional view of old age, which was closely linked to the social and biological understanding of ageing and the narrative of decline—a stage marked by losses and decay [8]—has been challenged through the emergence of the idea of rejuvenation, happiness, well-being and prolongevity in the new generation of older adults, with a special focus on the model of successful ageing, which has greatly altered the definitions and understandings of the process of growing old.

## 2. Psychological Well-Being and Successful Ageing

A significant body of scholarship shows that physical and psychosocial well-being in later life is an integral part of ageing well, which includes various individual, sociocultural, economic and environmental aspects [15]. High levels of subjective well-being, such as feelings of happiness, enjoyment, personal aspirations and achievement can increase physical health and longevity [16], which leads to the conclusion that happier people live longer and better lives [17]. In fact, “wellness is defined as the experience of happiness or satisfaction, challenges that require effort to overcome them, and the achievement of valuable goals” [18] (p. 1). In other words, our ability to overcome stress is one of the primary determinants of longevity and quality of life. However, stress levels accumulate progressively as we grow older, and the inability to manage life adversities and stressful situations may negatively affect our health and life quality in old age [7]. That is to say, by living longer, we become more exposed to the possibility of ageing with disability and chronic age-related diseases, which can negatively affect our quality of life and sense of happiness and well-being. Chronic daily stress, especially, “can lead to physical and cognitive disorders that hurt the health and well-being of older adults, exerting a negative influence on successful ageing” [7] (p. 3). Therefore, the questioning and negotiation of stress and the pursuit of fulfilling activities are especially important when looking at older people’s well-being, which is often defined by their ability to adjust to the ideal model of successful ageing.

The modern concept of ‘successful ageing,’ as an antithesis to the narrative of decline [8], emerged in the US in the second half of the 20th century [19,20]. Applied as a positive model to measure life satisfaction, it aimed to replace negative constructions of age and emphasise positive aspects of the process of growing old [20] (p. 26). According to this model, old age is portrayed as an enriching period in life that opens up an umbrella of possibilities that encompass active sexual lives, youthful looks and happy, healthy and secure living [19,20,21,22,23,24,25]. The images of the older population as passive and ill have also been eclipsed by positive representations of the retirement stage, compared to “an extension of the short vacation” [26] (p. 4). By the same token, Andrew Blaikie adds that “the hedonistic joys of leisured freedom” and new adventures “on the road in recreational vehicles” are promoted as all-desired active and pleasurable pastimes of older people [11] (p. 15). 

This anti-ageing ideal “was later crystallized” in the works of social scientists, John Rowe and Robert Kahn (1987, 1997, 1998) [27] (p. 26). Rowe and Kahn’s defined model of successful ageing focused on three main standards: the avoidance of disease and disability, high levels of cognitive and physical functioning and active social engagement. Active engagement in life meant being connected to people and productive activities. In their work *Successful Aging* (1998), Rowe and Kahn claimed that people can adhere to the ideals of successful ageing through proper life choices and individual responsibility and effort: “[o]ur main message is that we can have a dramatic impact on our own success or failure in ageing. Far more than is usually assumed, successful ageing is in our own hands” [27] (p. 18). In order to age according to this model, people had to meet all three criteria, meaning that those who did not were considered as ageing unsuccessfully and having no control over their lives. All in all, the main idea behind this model is that as long as older people maintain healthy lifestyles, are vibrant, energetic and enthusiastically participate in social circles and leisure, they are ageing in a correct and expected way [11,19,20]. 

If Rowe and Kahn’s idea of successful ageing is focused on people’s ability to manage their lives and be actively engaged in society, Paul and Margret Baltes’ proposed model is based on psychosocial theory of successful ageing as a lifelong process [28]. The scholars regard the process of ageing as a selective optimization with compensation model (SOC), which involves three elements: 1. selection: selecting and focusing one’s efforts into areas of high priority; 2. optimization: engaging in behaviours that enrich one’s physical and mental abilities, and 3. compensation: using psychological and technological strategies to enhance one’s quality of life. The three elements interplay with each other with the aim to optimize and enrich one’s subjective well-being and positive emotions and to minimize undesired outcomes and feelings of unhappiness or loneliness [28,29].

Disseminated through various social media and print, the model of success in old age continues to influence policies, research, public health and social trends. It has gained in popularity also because it presented a rethinking of the narrative of decline [8] and emphasised growth and new possibilities [24] (p. 33). It is also important to note that thanks to redefinitions of old age, there has been a significant reduction of ageism and a greater inclusion at work, better social support, creation of age-friendly cities and life-long learning and leisure opportunities. For instance, mobile technologies offer an array of possibilities to measure one’s physical activity, behaviour and sleep, which helps to prevent illness and to stay healthy and fit in older age [30]. All in all, because of rapidly changing demographic trends, the meanings of old age have become less defined and more negotiable in sociocultural representations of ageing. Older people are no longer portrayed as passive and dependant on their children, but as active and energetic individuals who go beyond age-related categorization and modes of life.

However, successful ageing has also received a lot of criticism as being too narrowly-defined, missing subjective perspectives of ageing, focusing too much on personal accountability, discipline and moral responsibly in order to age ‘well’ and lacking a more holistic approach [31] (pp. 480–481). That is to say, although this discourse offers new possibilities to older people, not all ageing subjects are able to keep up with its high requirements and pressures. In other words, even though the idea of successful ageing, along with related concepts of ‘positive’, ‘healthy’, ‘happy’ and ‘active’ ageing, has been embraced with positivism, the imposition of the privileged model of success in old age ignores the diversity of the experiences of ageing. Instead of liberating individuals who enter the second half of their lives, the successful ageing discourse, closely intersected with neo-liberal rationality and capitalist ideology, imposes new ways and regulations on ageing subjects [19,20,21,24,31]. In fact, the adjective ‘successful’ has itself proven problematic because it divides older people into two categories: winners and losers; one cannot “call someone *unsuccessful* merely because he or she is disabled or diagnosed with diabetes” [32] (p. 728, emphasis in original). 

Chris Phillipson [33] has pointed out that the collective responsibilities of the state have been shifted onto individuals, who struggle to keep up with anti-ageing ideals in a neo-liberal global context. According to the scholar, the successful ageing discourse was valid and useful in the 1990s, but taking into account the current globalized climate, harshly affected by economic crises, insecurity in workplaces, the rollback of public pensions and the dismantling of the welfare state and social protection, the idea of successful ageing loses ground. Instead of granting empowerment and new opportunities, it may lead to the further exploitation of older people, which is manifested by precarious living conditions and inequality. These aspects are much more visible in those who are housebound, have cognitive and physical frailties and lack access to new technologies and medical advances. Such structural inequalities can constitute life-long disadvantages and may even contribute to the victimization or further exclusion of those older individuals who do not or cannot adjust to the mainstream standards of growing old. 

Katz also observes that the focus on active citizenship creates new mandates for older people to be “retirement-ready and fit” [34] (p. 148). In fact, according to Katz, the idea of remaining forever functional and youthful in later life can be seen as a business strategy or as “a panacea for the political woes of the declining welfare state and its management of so-called risky populations” [34] (p. 147). The same thoughts are expressed by Chris Gilleard, who argues that the “desire for a long (and disability free) life is legitimized as part of good governance,” which exposes the alliance between the state and increasingly growing anti-ageing business [35] (p. 82). Toni Calasanti and Neal King follow the same line of thought by stating that “[s]uccessful ageing means not ageing and not being old because our constructions of old age contain no positive content” [36] (p. 7). In other words, the model of successful ageing “has acted as a vortex of professional, political and commercial interests” and, commercially, “has resonated with consumerist discourses that proffer an expanding horizon of anti-ageing goods and services” [36] (pp. 2–3). However, even if the model of successful ageing is a hard-achievable ideal, it has been widely embraced in popular culture and has successfully drifted into anti-ageing focused enterprises, promising everlasting happiness and life satisfaction in older age [24,34,36]. It is well-integrated into Western thought and widely visible in advertising, politics, the media and social circles, with a special focus on the anti-ageing industry, sexual performance, and even mental optimization. 

## 3. The Anti-Ageing Industry, Sexual Optimization and Mental Performance

Based on the model of successful ageing, the anti-ageing industry spreads a common message that an ageing body must be reshaped, remade, rejuvenated and adjusted to social expectations, which emphasises beauty and youth as the ultimate aims to which every ageing individual should aspire [37,38]. Although this discourse puts new pressures onto ageing individuals, especially in terms of bodily image, it also shows that the fear of ageing should not be paralysing because the signs of ageing can be efficiently effaced from one’s body. Personal responsibility of self-care, moral codes and living up to social expectations are often justified by scientific and medical advances that promote the idea that having youthful looks guarantees healthier, better and happier lives in later life [12,24,34,38,39]. Michelle Hannah Smirnova observes that references to doctors help to legitimize advertisements that suggest that the technologies used to make the product have been “innovative, unique and akin to other patent protected scientific technologies such as pharmaceutical medical treatments” [39] (p. 1241). However, a panoply of these advertisements “do not specify the role the doctor played in the development or use of the product. This might serve to imply a more direct connection between doctors and the cosmeceutical than that which actually exists” [39] (p. 1241). Ageing people are offered a variety of practices that are said to help to improve their looks and are told that if they can control their bodies, they can also overcome their fear of ageing. In fact, anti-ageing products are not presented as tools that ameliorate external looks but are promoted as necessary items for happiness and health reasons, which legitimises the role of aspirational medicine and the anti-ageing discourse [12,24,34,38,40]. Anti-ageing cosmetics and other means of rejuvenation are now presented as tools to improve one’s health and the quality of life, similar to dieting, which is now endorsed by the notion of healthy nutrition and avoidance of serious diseases, rather than for aesthetic reasons. For instance, the skin, whose primarily function is to regulate body temperature via sweat and to protect from microorganisms and radiation, is perceived from an aesthetic point of view, aimed at keeping a youthful appearance [39] (p. 1243). In fact, the very term ‘cosmeceutical’ is a combination of the words ‘cosmetic’ and ‘pharmaceutical,’ which implies that ageing is a disease that must be treated and fixed by using a great array of now available curative anti-ageing methods, which can be employed by whoever is willing to take care of his/her general well-being [41] (p. 14).

Ageing individuals are taught that they have a fundamental right to their health and well-being and are eligible to “‘age better’ by ‘not being old’” [12] (pp. 145–146). In fact, anti-ageing medicine can be seen as a social movement or revolution whose aim is to show that ageing can be treated by the aid of innovations in biotechnology [42]. It is regarded as a tool that will soon “offer the ability to both add years to life and life to years” [42] (p. 643). Mykytyn states that “[c]onstructing anti-ageing as not-an-insurance product is a way of separating it from mainstream biomedical practice, which may not only be financially advantageous to practitioners but also reinforces the revolutionary nature of anti-ageing medicine” [42] (p. 649). The promoters of this ‘revolution’ in biomedical treatment are exempt from total responsibility for the health and well-being of their (potential) patients, as well as their health insurance [42] (p. 649). Expensive anti-ageing products and treatments must be purchased by patients/customers themselves, since they are not covered by insurance companies. This observation again points to the existence of neo-liberalist practices with the focus on individual responsibility in terms of one’s pursuit of happiness and well-being in later stages of life.

Additionally, in the successful ageing discourse, consumption of anti-ageing products is seen as an indicator of one’s morality and health; the more an aged person acquires, the healthier and happier he/she becomes. As Calasanti and King note, the visible physical signs of ageing are seen as markers of “personal goodness” and “[f]ailure to appear healthy permits others to stigmatize a person as unfit” [36] (p. 195). In fact, highly innovative technologies of preventive medicine, in tandem with the pharmaceutical industry and anti-ageing proponents, go as far as to create an illusion that people age and die not because it is a natural part of human nature, but because medical advances have not yet found the means to cure the diseases people age and die from [43]. Instead of promoting the acceptance of the natural process of ageing, aesthetic practitioners reinforce ageist ideas about the undesirability of older bodies and the need to reshape and to rejuvenate them [12,20,38,40,41]. 

Apart from the focus on bodily rejuvenation and the consumption of anti-ageing products, the model of successful ageing highlights sexual optimization in later life as a way towards a happy and fulfilled old age. That is to say, even though the successful ageing discourse allows for more freedom of sexual expression and helps to undo the stereotypes of older individuals seen as asexual and uninterested in romantic relations, it may also lead to social stratification and even exclusion of those who are not interested in having sex and/or cultivating intimate relationships in later life [12,36]. High pressures to maintain one’s youthful looks, mainly addressed to women in order to be ‘visible’ and sexualy appealing, have also been extended to men who are expected to be sexually active and adjust to new hyper masculine roles within the normative assumptions of gender and sexuality [23,36,44,45]. Those men who suffer from sexual dysfunction and/or do not use pharmaceutical drugs, can develop feelings of inadequacy and unhappiness because of their inability to adapt to the expected ideals of masculine potentiality and youthful virility [23,36,44,45]. In other words, older people who show little interest in sexual activities are often seen as ‘unsuccessful ageing subjects’ or ‘problem persons’ and are advised to see a doctor or a sexologist [12,34]. As Gilleard and Higgs argue, we live in a society where “sex and the expression of one’s sexuality become social virtues and indicators of emotional physical and mental well-being. Sexual expression became a right to which all are entitled, to the point that those unable to access sexual partners […] are considered to have ‘unmet needs’ that health and social care services should at least consider, if not meet” [12] (p. 109).

Sandberg and Marshall, apart from criticizing the importance given to sexual performance in later years, also interrogate the dominant perceptions of heteronormativity [46]. They urge for the need to open up alternative ways of looking at body image and sexuality and shed light on a greater diversity of later lives beyond the model of successful ageing. As the scholars state, “heteronormativity and its promises of happiness constitute a powerful narrative that organizes dominant understandings of the good (later) life”, thus excluding the queer, inactive and physically- and mentally-disabled subjects [46] (p. 3). 

Furthermore, Katz also observes that not only the rejuvenated body or sexual activity in later life, but also memory and the brain, have become technologically quantified in a neo-liberal consumer culture [47]. According to this scholar, the successful ageing discourse has been expanded into the governance of human minds, often measured by the use of digital technologies such as brain scans. The ageing population is advised to use various innovative means, such as online brain-game performance or ‘bio-games’, in order to optimize their brain health and “cognitive fitness,” seen as an indicator of successful ageing and adaptation to societal expectations. Yet, time and again, ageing subjects are made responsible for their own cognitive performance, which is measured by brain sciences and “align[ed] to capitalist standards of productivity, efficiency and speed” [47]. Moreover, an increasing fear of dementia contributes to the rise of the promotion of digital anti-ageing-oriented innovations, which equate cognitive abilities with successful ageing and risk-management [47]. 

However, the equation of one’s looks with well-being and the desire to stop the very process of growing old are so internalized by individuals that “solutions to both internal and external symptoms of ageing, the concepts of health, youth and beauty are collapsed onto each other” [39] (p. 1239). Those who do not comply with the successful ageing ideals, do not use innovative self-care devices and do not take advantage of scientific and technological advances are often regarded as neglectful, decadent and irresponsible citizens [34,36,39]. In other words, those who do not try to adjust to anti-ageing ideals are often regarded as failures or “problem persons” that need to be re-educated for their *own* benefit [34] (p. 148). That successful ageing does not challenge ageism is also illustrated in Gilleard’s observation that “[s]uccessful old age is old age without old age” [35] (p. 82). Following the same idea, Sandberg states that “successful ageing should perhaps more rightfully be termed successful non-ageing or agelessness” and calls for more inclusive and realistic definitions of ageing [22] (p. 13). 

## 4. Alternative Understandings of Successful Ageing

Many scholars have merged various disciplines to offer complementary understandings of old age, and have suggested considering alternative models that offer a less normative, unrealistic and exclusionary vision of later life. Taking into account diverse cultural perspectives beyond the Western, white and middle class-based conceptualizations of ageing well can help to broaden our current gerontological ageing framework and provide more inclusive and supportive policies and practices [48]. For example, Sandberg proposes an alternative conceptualization of ageing, which she defines as ‘affirmative old age’ [22]. Through this model, the scholar aims to visualize late-life as a stage to affirm differences, empower ageing subjects and give them new options within a wide range of possibilities that go “from active to sedentary, [and] from sexually vibrant to sexually indifferent” [22] (p. 35). Affirmative old age, according to Sandberg, not only helps to rethink the concept of ageing and late-life but can also serve “as social critique in a culture that eradicates difference” [22] (p. 15). To her, the diversity of lived experiences shows that ageing cannot be framed within narrow binary understandings but opens up new meanings associated with the last stage in human life.

Other proposed models, such as harmonious ageing [49], resilient ageing [50,51] or balanced ageing [52] integrate a spiritual and transcendental notion of ageing with a special emphasis on Eastern philosophies. For instance, Jiayin Liang and Baozhen Luo’s [49] model of harmonious ageing puts special emphasis on cultural and ethnic diversity when examining ageing realities. The scholars criticize the dominant conceptual framework of successful ageing and claim that the successful ageing discourse is ageist in its nature because it overlooks the harmony between body and mind and imposes capitalist and consumerist pressures on the older population. Liang and Luo believe that, by promoting an image of a sexually active, functional and busy citizen, this discourse denies diversity and complexity to the uniqueness of old age and, instead, creates a new image of a good old age, enshrined with anti-ageing methods. The scholars also note that the ideals of ageing are bound to Western value systems and ways of life, which overshadow other experiences of ageing in the global context and the existence of other theories in todays’ gerontological research. According to Liang and Luo, the multiple Western-centred theories on ageing are too fragmented, and there are too many of them [49] (p. 327). Thus, the scholars challenge the cultural blindness of contemporary Western society and, instead of elaborating on new theories, they propose to define ageing as ‘harmonious ageing,’ inspired by the Yin-Yang philosophy. According to Eastern values, harmony implies the balance between body and mind, the equilibrium in everyday experiences and a sense of continuity and change in the life course. The Yin-Yang philosophy also highlights the autonomy and freedom of choice of an individual, and the maintenance of one’s health and spirituality through the unity of physical exercise and mental health, since it is believed that inner peace helps to keep the physical body healthy. Because of its holistic approach towards human nature, the harmonious model of ageing can be applied to both Western and non-Western societies and cultures. As these scholars state, “[h]armony is created by an ageing individual by constantly achieving a balance between the structural and the individual, advantages and disadvantages, change and continuity, inadequacy and abundance, etc. Furthermore, because of its emphasis on balance that is achieved based on differences, harmony is expressed through a less standardized language than is the Western notion of success” [49] (p. 331).

Liang and Luo argue that the emphasis on harmony “contribute[s] to cross-cultural gerontological research, education and communication,” as it involves both the complexity and flexibility of ageing experiences that extend beyond the ethnocentric Western values of old age [49] (p. 327).

The need to redefine Western-centred ideals of successful ageing is also expressed by Sharon Wray [53], who argues that the dominant notions of ageing have neglected the multiple experiences of growing old. Wray argues that the experiences and priorities of ageing individuals differ significantly depending on their backgrounds. According to her, social, economic, cultural, ethical, racial, gender and religious differences create richer understandings of the experience of growing older and reveal that there is a great range of possibilities beyond Western ethnocentric ideologies and lifestyles, which are not always accessible, needed or even wanted by older individuals. However, because of a lack of greater empirical research and examination of more subjective viewpoints, the diverse perceptions of the ageing process still remains “under theorised” and creates “a distinctly Western understanding of midlife” [53] (p. 32). Hence, for Wray, it is crucial to be more sensitive to the complex nuances of old age and move away from hegemonic Western-specific norms in order to have a broader and more diverse vision of ageing. 

Other researchers state that, in fact, it is impossible to propose one agreed upon normative definition of success in old age because every individual has his/her own perception of happiness and well-being [48]. That is to say, there is no one specific ideal to meet the needs, desires and experiences of older people and, at the same time, to take into account their sociocultural, historical, religious and generational differences, among other aspects. 

## 5. Conclusions

This article has interrogated the current perceptions of happiness, health and well-being in later life from an interdisciplinary age-studies perspective. It has argued that the successful ageing discourse, rather than liberating older individuals, can generate stress and create new pressures to adapt to the ideals of successful ageing. In other words, the perception of a happy and fulfilling old age in Western cultures seems to be measured in terms of youthful physical appearance, active engagement in live, good mental health, the absence of diseases, sexual activity and heterosexual desirability and one’s ‘success’ in the successful ageing discourse. If older individuals cannot meet the demands of the model of successful ageing, they are more likely to develop feelings of inadequacy and accumulate stress levels that have a negative effect on their health and the notion of happiness and well-being in old age. As Virpi Timonen observes, to age successfully and gracefully “is clearly an all-consuming, expensive project!” [24] (p. ix). However, the ideals of the successful ageing discourse are so integrated in the contemporary Western ‘measurement’ of the well-being and happiness of old people that the questioning of its benefits “would be considered unprofessional, if not heretical” [34] (p. 135). Therefore, it is of paramount importance to offer more inclusive and diverse understandings of growing older, taking into account the voices of older people themselves. In fact, ‘success’ is a very personal definition and subjective judgment, which is based on one’s social, religious and cultural backgrounds, upbringing and value system. In other words, what success is to one individual or culture can be failure to another [54] (p. 744). Instead of blaming older individuals with physical and cognitive disabilities, multiple chronic conditions or structural inequalities and subjecting them to moral judgments, neoliberal ideologies and hard-achievable ideals of the exclusionary models of successful ageing, we should promote and embrace our differences in old age. Alternative, holistic and more inclusive definitions of successful ageing could benefit healthcare professionals, politicians, stakeholders and researchers in the field of ageing, as well as ageing subjects themselves. In summary, unless ageing individuals raise their voices against being pressured into adopting the successful ageing expected lifestyles and start defining their own ageing identities, they will not be truly liberated and ‘happy’. 
